# Recorded body height and sex differences in lung-protective ventilation among adults with ARDS: an 11-year single-centre cohort study

**DOI:** 10.1186/s40560-026-00936-w

**Published:** 2026-08-31

**Authors:** Marius Huehn, Lea Willemsen, Matthias Manfred Deininger, Judith Huth, Jana C. Mossanen, Jens Spiesshoefer, Gernot Marx, Tim-Philipp Simon, Thomas Breuer

**Affiliations:** 1 Department of Intensive Care Medicine, Faculty of Medicine, RWTH Aachen University, Pauwelsstraße 30, 52074 Aachen, Germany; 2 Department of Anesthesiology, Faculty of Medicine, RWTH Aachen University, Pauwelsstraße 30, 52074 Aachen, Germany; 3 Department of Pneumology and Intensive Care Medicine, Faculty of Medicine, RWTH Aachen University, Pauwelsstraße 30, 52074 Aachen, Germany

**Keywords:** Acute respiratory distress syndrome, Lung-protective ventilation, Tidal volume, Predicted body weight, Body height, Mechanical power, Sex differences, Health-care disparities, Surgical intensive care

## Abstract

**Background:**

Lung-protective ventilation (LPV) reduces mortality in ARDS, but adherence remains incomplete and women and shorter patients disproportionately receive tidal volumes above predicted-body-weight (PBW)-based targets. The relative associations of recorded sex and body height with this disparity remain uncertain.

**Methods:**

Retrospective single-centre cohort study (January 2013 to December 2023, RWTH Aachen University Hospital surgical ICU). Adults (≥ 18 years) with Berlin-definition ARDS receiving mechanical ventilation for ≥ 72 h were included. Patient-level adherence to lung-protective targets was described, and repeated-measure PBW-normalised tidal volume and mechanical power were analysed with linear mixed-effects models containing a patient-level random intercept and fixed effects for recorded sex, centred recorded height, age, BMI, SAPS II, SOFA, ARDS aetiology, linear relative ICU day and the lowest same-day PaO₂/FiO₂ ratio.

**Results:**

Among 798 adults (263 women [33.0%]), unadjusted analyses showed that women received higher tidal volumes per kg PBW than men (median, 7.6 vs. 6.8 mL/kg PBW; p < 0.001), with lower VT adherence (55.3% vs. 76.1%; p < 0.001), higher plateau and driving pressures, and higher PBW-normalised mechanical power. In 527 patients contributing 58,802 observations, the simultaneous adjusted VT model yielded a female-sex coefficient of 0.051 mL/kg PBW (95% CI, −0.273 to 0.376; p = 0.756), whereas recorded height was inversely associated with VT/kg PBW (β per cm, −0.0342; 95% CI, −0.0498 to −0.0185; p < 0.001); the sex-by-height interaction was not supported (p = 0.971). In the corresponding mechanical-power model (410 patients; 44,725 observations), the female-sex coefficient was −0.0158 J·min⁻^1^·kg PBW⁻^1^ (95% CI, −0.0512 to 0.0196; p = 0.384).

**Conclusions:**

Unadjusted analyses showed lower lung-protective ventilation adherence in women. In the simultaneous adjusted model, recorded height, but not recorded sex, was independently associated with VT/kg PBW. Because PBW incorporates both sex and height and recorded height was not validated against a reference measurement, the study does not establish mediation or measurement error as the underlying mechanism. Standardised direct height measurement and PBW-based decision support warrant prospective evaluation.

**Supplementary Information:**

The online version contains supplementary material available at https://doi.org/10.1186/s40560-026-00936-w.

## Background

Lung-protective ventilation (LPV) for the acute respiratory distress syndrome (ARDS)—low tidal volume (VT) referenced to predicted body weight (PBW), plateau pressure (Pplat) ≤ 30 cm H₂O and driving pressure (DP) < 15 cm H₂O—has been guideline-endorsed standard of care since the Acute Respiratory Distress Syndrome Network trial reported an absolute mortality reduction with low-VT ventilation more than two decades ago [1–3]. Despite this evidence, international observational cohorts continue to report that only 60 to 75% of eligible patients receive guideline-concordant tidal volumes [4, 6, 7], with women and shorter patients disproportionately receiving non–lung-protective ventilation [8–10]. The addition of driving pressure as an outcome-relevant target has further sharpened the relevant thresholds but has not closed the adherence gap [5].

Tidal volume in LPV protocols is referenced to PBW, which is a deterministic function of recorded body height and sex through the Devine formula [1, 11, 12]. Consequently, the same absolute tidal volume yields a higher VT/kg PBW in shorter patients and, at the same recorded height, in women. Accurate height documentation is therefore important for PBW-based ventilator settings. Previous studies have shown that visually estimated height may be inaccurate and may expose shorter patients to larger tidal volumes than intended [13, 14]. However, a recorded association with body height does not by itself demonstrate that inaccurate height acquisition is the mechanism underlying a sex-associated ventilation difference.

Three gaps motivated this study. First, the temporal course of sex-associated differences in LPV delivery has rarely been evaluated across more than a decade of routine practice. Second, the extent to which the association is attenuated when patients are compared across recorded height ranges remains uncertain. Third, mechanical load expressed as PBW-normalised mechanical power (MP) has seldom been examined in this context, although normalised MP may capture a different aspect of ventilatory exposure than absolute MP [15–17].

We therefore aimed to (1) describe sex-associated differences in LPV delivery across 11 consecutive calendar years, (2) examine the simultaneous associations of recorded sex and recorded body height with PBW-normalised VT using multivariable and exploratory height-stratified analyses, and (3) evaluate sex-associated differences in absolute and PBW-normalised mechanical power. Because the method and accuracy of height acquisition were not validated against a reference measurement, analyses concerning body height were considered associative rather than causal.

## Methods

### Study design and reporting

This was a retrospective, single-centre, observational cohort study at the Department of Intensive Care Medicine of RWTH Aachen University Hospital, Aachen, Germany, covering January 1, 2013 through December 31, 2023. This report follows the Strengthening the Reporting of Observational Studies in Epidemiology (STROBE) statement for cohort studies. The study was approved by the Ethics Committee of the Medical Faculty of RWTH Aachen University (No. 23–381; January 15, 2024); the requirement for written informed consent was waived because only retrospective anonymised data were analysed. The study conformed to the Declaration of Helsinki.

### Participants

All adults (≥ 18 years) admitted between January 2013 and December 2023 with a primary or secondary International Statistical Classification of Diseases, Tenth Revision (ICD-10) code of J80.X were screened. The ICD-based screen preceded clinical adjudication of the Berlin criteria [18]. Inclusion required documented fulfilment of the Berlin definition and ≥ 72 h of mechanical ventilation in the ICU. Cases in which pulmonary oedema was considered primarily hydrostatic, including isolated heart failure without ARDS, were classified as not fulfilling the Berlin criteria during adjudication and were excluded at this stage.

### Data sources

Clinical data were retrieved from two integrated electronic patient-record systems: Medico (version 29.00.02.00; CompuGroup Medical SE & Co. KGaA, Koblenz, Germany) and IntelliSpace Critical Care and Anesthesia (ICCA, version J.05.01; Koninklijke Philips N.V., Amsterdam, the Netherlands). At each new hospital admission, height documentation was mandatory. A new value was entered into Medico by the assessing anaesthesiologist, most often during the preoperative anaesthetic assessment, and was not automatically carried forward from previous admissions. The accompanying structured field classified the source as “self-reported” or “determined by physician.” The latter category did not distinguish direct measurement from visual estimation. A proxy could provide the information when the patient was unable to do so, but proxy reports were not coded separately. At ICU admission, the height value and acquisition category were transferred to ICCA. This process remained unchanged from 2013 through 2023. The dataset did not contain a reference measurement against which the recorded height could be validated.

### Exposures and ventilation parameters

Primary exposures were recorded female versus male sex (retrieved from administrative records) and recorded body height, analysed continuously and in prespecified strata (< 165, 165–175 and > 175 cm). All evaluated ventilator values were derived from controlled mechanical ventilation, identified through structured mapping of ventilator-mode labels. Pplat and positive end-expiratory pressure (PEEP) were extracted directly; driving pressure was calculated as Pplat − PEEP. VT was normalised to PBW using the Devine formula adopted by the ARDS Network [1, 11]. For the primary mechanical-power analysis, the same simplified Becher equation for pressure-controlled ventilation was applied uniformly: 0.098 × respiratory rate × VT in litres × (ΔPinsp + PEEP), where ΔPinsp was peak inspiratory pressure minus PEEP [19–21]. In a formula sensitivity analysis, 166 patients had the variables required for the Gattinoni formulation; 146 of these also had temporally overlapping estimates from both methods, and complete covariate data at identical valid time points were available for 85 patients. The Gattinoni equation was 0.098 × RR × {VT^2^ × [½ × ELrs + RR × (1 + I)/(60 × I) × Raw] + VT × PEEP}, where RR is expressed in breaths/min, VT in litres, respiratory-system elastance (ELrs) in cm H₂O/L, airway resistance (Raw) in cm H₂O·s/L, I is the dimensionless inspiratory-to-expiratory ratio, and pressures are expressed in cm H₂O. The factor 0.098 converts cm H₂O·L/min to J·min⁻^1^. Formula-specific models and a model of the paired Gattinoni-minus-Becher difference used the same fixed-effect specification.

### Outcomes

Ventilation outcomes comprised repeated-measure VT/kg PBW, which was used in the primary multivariable model, and patient-level adherence to the tidal-volume target of VT ≤ 8 mL/kg PBW. Additional ventilation outcomes were Pplat ≤ 30 cm H₂O, driving pressure < 15 cm H₂O, absolute VT, and absolute and PBW-normalised mechanical power. For each patient, the proportion of controlled-ventilation measurements meeting each threshold was calculated. Mechanical power was analysed as a continuous secondary exposure metric; no adherence threshold for mechanical power was defined. Secondary clinical outcomes included ICU and hospital mortality, lengths of stay, duration of mechanical ventilation and prespecified ICU complications.

### Statistical analysis

Analyses were performed in R version 4.3.3 within RStudio version 2025.05 [22, 23]. Linear mixed-effects models were fitted using lme4 [24], with Satterthwaite p-values obtained using lmerTest and 95% Wald confidence intervals. Patient identifier was included as a random intercept. Relative ICU day was defined as elapsed time since ICU admission (day 0 through day 29) and was entered as a continuous linear covariate in the adjusted VT and mechanical-power models, including the absolute-outcome, interaction and height-stratified variants. Calendar year was analysed separately in the prespecified sex-by-calendar-year interaction model.

The single SOFA value available for each patient was defined as the maximum score within the first 24 h after ICU admission. The primary multivariable model for PBW-normalised VT included recorded sex, recorded height centred at the mean of the VT complete-case analysis sample (174.1 cm), age, BMI, SAPS II, SOFA, ARDS aetiology, relative ICU day and the lowest same-day PaO₂/FiO₂ ratio as fixed effects. Observations without a same-day PaO₂/FiO₂ value were excluded. Analyses used complete cases. A sex-by-height interaction was assessed in a separate model, and variance inflation factors were calculated for the fixed-effects design matrix. Prespecified exploratory models were fitted within each height stratum. A sensitivity analysis used reconstructed absolute VT in mL as the outcome with the same covariate set. A further sensitivity model additionally included the documented height-source category (physician-determined versus self-reported).

Mechanical-power models used the same fixed-effect set and complete-case approach as the VT models, with recorded height centred at the mean of the mechanical-power complete-case analysis sample (174.46 cm) and a patient-level random intercept. Models were fitted for absolute and PBW-normalised mechanical power and separately within the prespecified height strata. The primary analysis used the same Becher pressure-controlled-ventilation equation for every included patient. The restricted formula sensitivity analysis applied identically specified models to the Gattinoni estimate, the Becher estimate and their paired difference at corresponding time points.

ICU mortality was assessed in an exploratory patient-level complete-case logistic-regression model containing recorded sex, age, SAPS II and SOFA. Outliers were handled using the prespecified median-absolute-deviation procedure [25].

## Results

### Cohort

**Fig. 1:**
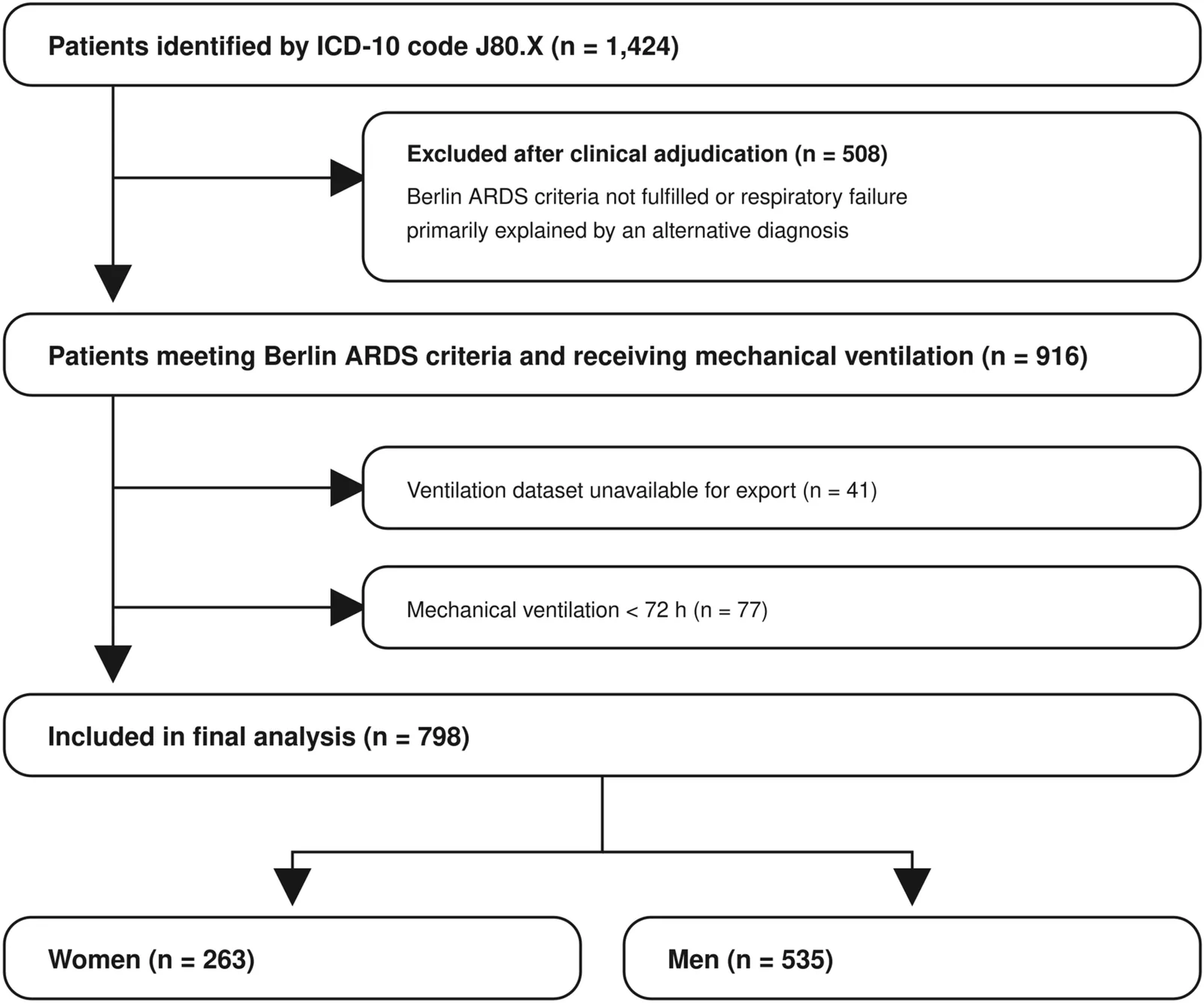
Study flow diagram. Of 1,424 patients identified by ICD-10 code J80.X, 508 were excluded after clinical adjudication because the Berlin ARDS criteria were not fulfilled or respiratory failure was primarily explained by an alternative diagnosis. Of 916 patients meeting the Berlin ARDS criteria and receiving mechanical ventilation, 41 had no exportable ventilation dataset and 77 were ventilated for < 72 h, leaving 798 patients (263 women and 535 men) in the final analysis

Of 1,424 patients identified by the ICD-based screen between 2013 and 2023, 798 fulfilled all eligibility criteria and were included (Fig. 1). The cohort included 263 women (33.0%) and 535 men (67.0%); median [IQR] age was 59 [48–69] years. Women were shorter and had lower absolute body weight than men. Median BMI was 28.3 [24.2–36.2] kg/m^2^ in women and 27.7 [24.7–31.8] kg/m^2^ in men (p = 0.053); obesity was present in 110 women (41.8%) and 177 men (33.1%; p = 0.016). The documented height source was self-reported for 386/798 patients (48.4%) and determined by a physician for 412/798 (51.6%). The latter category did not distinguish direct measurement from visual estimation. The complete-case height-stratified analyses included 87 patients below 165 cm (14 men and 73 women), 213 at 165–175 cm (135 men and 78 women), and 227 above 175 cm (215 men and 12 women). Other baseline and clinical characteristics are shown in Table 1.

**Table 1:** Patient characteristics, ICU complications and outcomes by sex

Characteristic	Female (n = 263)	Male (n = 535)	p-value
Age, median [IQR], years	58 [48–69]	60 [49–70]	0.25
Height, median [IQR], cm	165 [160–170]	180 [173–183]	< 0.001
Weight, median [IQR], kg	80 [65–95]	90 [75–100]	< 0.001
Source of documented height, n (%)			
Self-report	133 (50.6)	253 (47.3)	
Determined by physician	130 (49.4)	282 (52.7)	
BMI, median [IQR], kg/m^2^	28.3 [24.2–36.2]	27.7 [24.7–31.8]	0.053
Obesity (BMI ≥ 30 kg/m^2^), n (%)	110 (41.8)	177 (33.1)	0.016
SAPS II at admission, median [IQR]	40 [32–47]	40 [32–47]	0.83
SOFA, maximum within the first 24 h after ICU admission, median [IQR]	10 [7–12]	10 [7–12]	0.81
ARDS severity (Berlin), n (%)			
Mild	14 (5.3)	32 (6.0)	
Moderate	106 (40.3)	202 (37.8)	
Severe	133 (50.6)	290 (54.2)	
Mean PEEP per patient, median [IQR], cm H₂O	9 [8–10]	10 [8–11]	< 0.001
Comorbidities, n (%)			
Hypertension	126 (47.9)	271 (50.7)	0.47
Peripheral arterial disease	24 (9.1)	79 (14.8)	0.03
Diabetes mellitus type 2	63 (24.0)	139 (26.0)	0.54
Chronic kidney disease	20 (7.6)	50 (9.3)	0.41
COPD	48 (18.3)	68 (12.7)	0.04
Asthma	13 (4.9)	7 (1.3)	0.002
Nicotine use	67 (25.5)	129 (24.1)	0.67
Alcohol use	23 (8.7)	82 (15.3)	0.01
ARDS aetiology, n (%)			
Trauma	53 (20.2)	111 (20.7)	0.84
Bacterial infection	236 (89.7)	446 (83.4)	0.02
Fungal infection	1 (0.4)	13 (2.4)	0.04
Viral infection	2 (0.8)	22 (4.1)	0.009
Unknown infection	7 (2.7)	45 (8.4)	< 0.001
ICU complications, n (%)			
Delirium	96 (36.5)	230 (43.0)	0.08
Urinary tract infection	62 (23.6)	80 (15.0)	0.003
Acute kidney failure	170 (64.6)	336 (62.8)	0.61
Dialysis required	120 (45.6)	238 (44.5)	0.76
Acute liver failure	39 (14.8)	68 (12.7)	0.41
Sepsis	254 (96.6)	523 (97.8)	0.33
Septic shock	253 (96.2)	517 (96.6)	0.75
Tracheotomy	201 (76.4)	425 (79.4)	0.33
Pleural effusion	209 (79.5)	440 (82.2)	0.34
Re-intubation	66 (24.5)	131 (25.1)	0.92
Outcomes			
ICU mortality, n (%)	107 (40.7)	218 (40.7)	0.99
Hospital mortality, n (%)	109 (41.4)	219 (40.9)	0.89
ICU length of stay, median [IQR], days	33 [18–54]	31 [18–53]	0.93
Hospital length of stay, median [IQR], days	41 [22–68]	39 [22–61]	0.33
Duration of mechanical ventilation, median [IQR], hours	560 [319–900]	585 [324–956]	0.81

Data are presented as median [IQR] or n (%). Statistical comparisons used Wilcoxon rank-sum tests or χ^2^ tests, as appropriate. Percentages for ARDS severity do not sum to 100% because of missing PaO₂/FiO₂ values. BMI body mass index, COPD chronic obstructive pulmonary disease, ICU intensive care unit, IQR interquartile range, PEEP positive end-expiratory pressure, SAPS II Simplified Acute Physiology Score II, SOFA Sequential Organ Failure Assessment

### Lung-protective ventilation adherence by sex

**Fig. 2:**
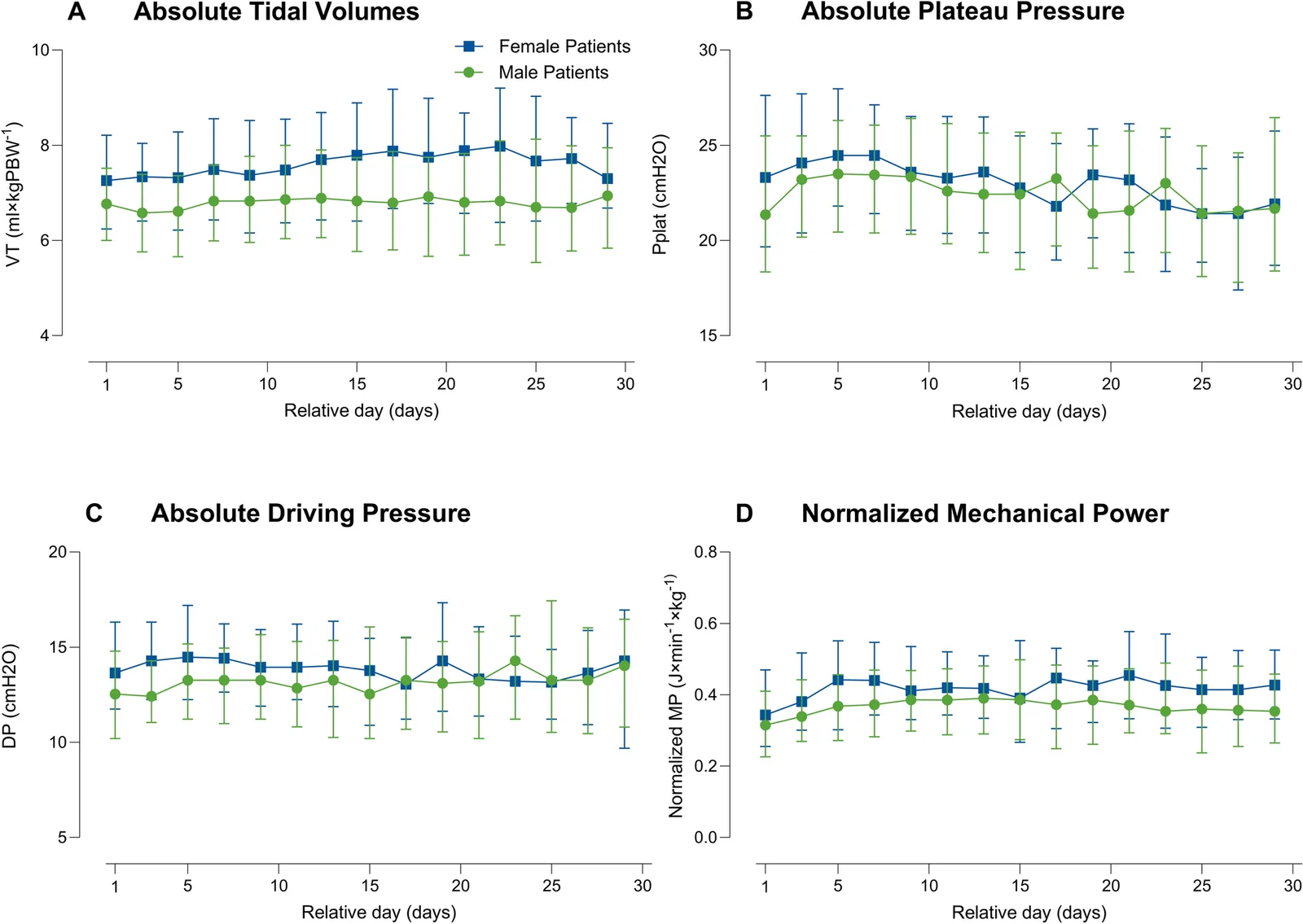
Sex-specific ventilation parameters during the first 30 days of ICU stay. **a** Tidal volume per kilogram of predicted body weight (PBW). **b** Plateau pressure. **c** Driving pressure. **d** PBW-normalised mechanical power. Error bars indicate 95% confidence intervals

During the first 30 days after ICU admission, women received higher PBW-normalised tidal volumes than men (median [IQR], 7.6 [6.7–8.4] mL/kg PBW vs. 6.8 [6.2–7.5] mL/kg PBW; p < 0.001), with a lower patient-level VT-adherence fraction (55.3% [34.5–78.0%] vs. 76.1% [58.8–88.5%]; p < 0.001) (Table 2; Fig. 2). The disparity was also observed at the more conservative 4 to 6 mL/kg PBW threshold (Additional file 1: eFig. 3). Plateau pressure (23.9 [21.4–26.6] cm H₂O vs. 22.5 [20.2–25.4] cm H₂O; p < 0.001) and driving pressure (14.0 [12.3–15.9] cm H₂O vs. 12.9 [11.2–14.9] cm H₂O; p < 0.001) were both higher in women. The patient-level plateau-pressure-adherence fraction was similarly high in both sexes but statistically differed (median [IQR], 100% [89.3–100%] vs. 100% [95.7–100%]; p = 0.023), whereas the patient-level driving-pressure-adherence fraction (< 15 cm H₂O) was substantially lower in women (45.7% [8.9–83.2%] vs. 65.1% [21.2–95.9%]; p < 0.001). PBW-normalised mechanical power was higher in women (0.41 [0.32–0.51] J·min⁻^1^·kg PBW⁻^1^ vs. 0.37 [0.30–0.45] J·min⁻^1^·kg PBW⁻^1^; p < 0.001), although absolute MP was higher in men (22.8 [18.8–27.5] vs. 27.1 [21.8–32.3] J·min⁻^1^), reflecting the larger PBW that the normalisation removes.

**Table 2:** Ventilation parameters during the first 30 days of ICU stay, by sex

Parameter	Female, n	Female, median [IQR]	Male, n	Male, median [IQR]	p-value
VT, mL/kg PBW	257	7.6 [6.7–8.4]	513	6.8 [6.2–7.5]	< 0.001
VT lung-protective fraction (≤ 8 mL/kg PBW), % [IQR]	257	55.3 [34.5–78.0]	513	76.1 [58.8–88.5]	< 0.001
Plateau pressure, cm H₂O	248	23.9 [21.4–26.6]	486	22.5 [20.2–25.4]	< 0.001
Pplat lung-protective fraction (≤ 30 cm H₂O), % [IQR]	248	100 [89.3–100]	486	100 [95.7–100]	0.023
Driving pressure, cm H₂O	218	14.0 [12.3–15.9]	436	12.9 [11.2–14.9]	< 0.001
DP lung-protective fraction (< 15 cm H₂O), % [IQR]	218	45.7 [8.9–83.2]	436	65.1 [21.2–95.9]	< 0.001
Mechanical power, absolute, J·min⁻^1^	197	22.8 [18.8–27.5]	425	27.1 [21.8–32.3]	0.001
Mechanical power, PBW-normalised, J·min⁻^1^·kg PBW⁻^1^	197	0.41 [0.32–0.51]	425	0.37 [0.30–0.45]	< 0.001

Data are presented as median [IQR]. Lung-protective fractions were computed at the patient level (proportion of controlled-ventilation measurements meeting the threshold per patient) and compared between sexes using Wilcoxon rank-sum (Mann–Whitney U) tests on the patient-level fractions. IQR interquartile range, PBW predicted body weight, VT tidal volume, Pplat plateau pressure, DP driving pressure

**Fig. 3:**
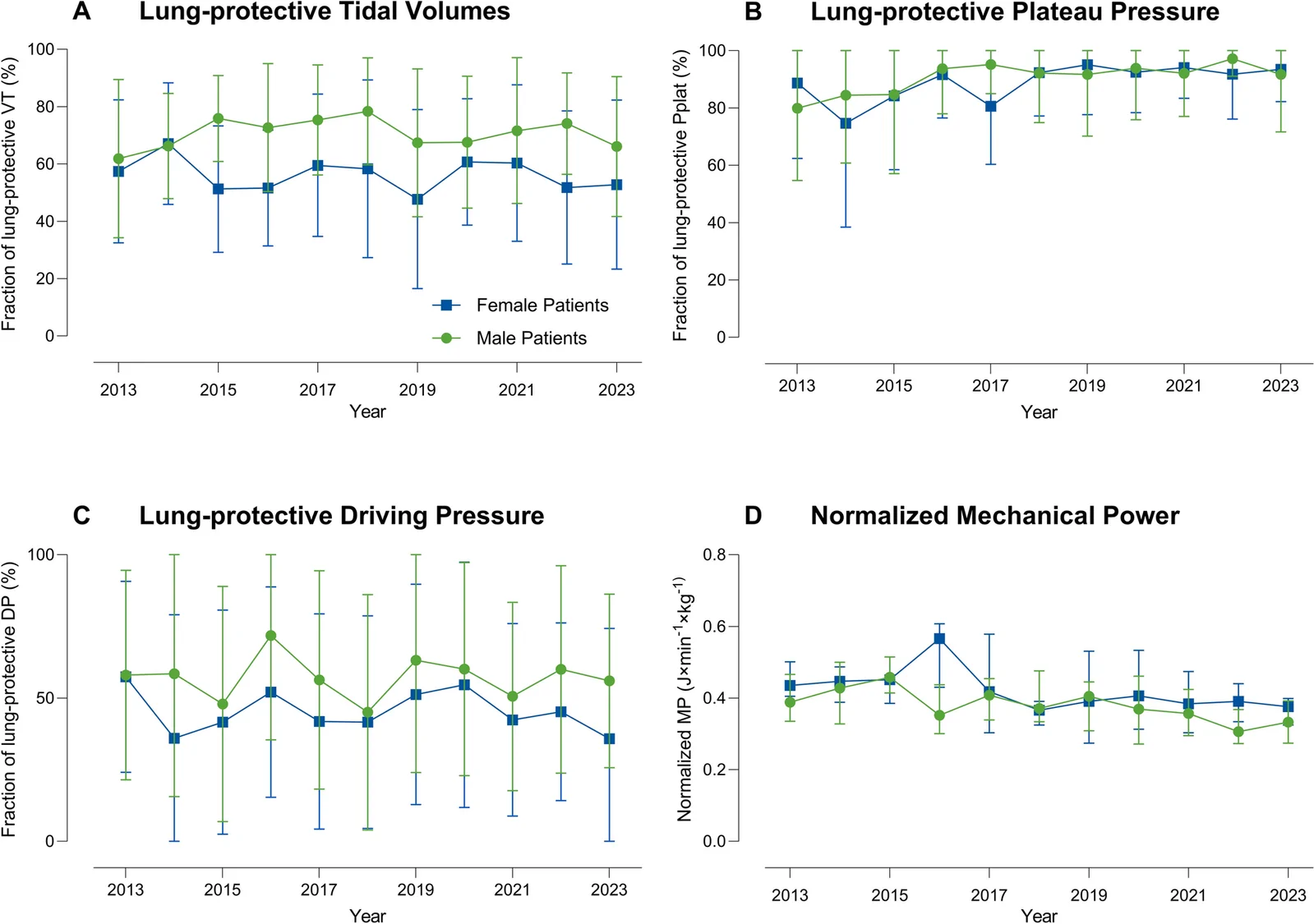
Year-by-year lung-protective ventilation adherence, 2013 through 2023. **a** Percentage of measurements within tidal volume 4 to 8 mL/kg PBW. **b** Percentage within plateau pressure ≤ 30 cm H₂O. **c** Percentage within driving pressure < 15 cm H₂O. **d** PBW-normalised mechanical power. Error bars indicate 95% confidence intervals

The year-by-year analysis from 2013 through 2023 showed a sex-associated difference throughout the observation period (Fig. 3). In the prespecified sex-by-calendar-year model, no interaction was detected (β = −0.003 mL/kg PBW per year; 95% CI, −0.09 to +0.09; p = 0.96). This analysis found no evidence of temporal narrowing or widening, but the confidence interval does not establish equivalence or prove that the difference was constant over time.

### Height-stratified analysis

Of 163,276 eligible VT observations, 82,595 (50.6%) had a same-day PaO₂/FiO₂ value; after complete-case exclusion for all model covariates, the primary model included 58,802 observations from 527 patients. With recorded sex and height entered simultaneously, the female-sex coefficient was 0.051 mL/kg PBW (95% CI, −0.273 to 0.376; p = 0.756), whereas each additional centimetre of recorded height was associated with −0.0342 mL/kg PBW (95% CI, −0.0498 to −0.0185; p < 0.001). The sex-by-height interaction coefficient was 0.0006 mL/kg PBW per cm (95% CI, −0.0331 to 0.0344; p = 0.971). Variance inflation factors were all ≤ 1.75.

In the height-stratified models, female-sex coefficients were −0.055 mL/kg PBW below 165 cm (87 patients: 14 men and 73 women; 95% CI, −0.93 to 0.82; p = 0.902), 0.067 mL/kg PBW at 165–175 cm (213 patients: 135 men and 78 women; 95% CI, −0.32 to 0.46; p = 0.737), and −0.109 mL/kg PBW above 175 cm (227 patients: 215 men and 12 women; 95% CI, −0.87 to 0.65; p = 0.779). The absolute-VT sensitivity model yielded a female-sex coefficient of −30.1 mL (95% CI, −51.9 to −8.4; p = 0.007) and a height coefficient of 3.98 mL per cm (95% CI, 2.93 to 5.03; p < 0.001). The documented height-source category (physician-determined versus self-reported) was not independently associated with VT/PBW (β = −0.087 mL/kg PBW; 95% CI, −0.322 to 0.147; p = 0.465), and its inclusion did not materially change the sex or height coefficients (Additional file 1: eTable 2D).

### Secondary outcomes and sensitivity analyses

Unadjusted ICU mortality was 40.7% in women and 40.7% in men; hospital mortality was 41.4% and 40.9%, respectively. In the exploratory complete-case logistic model (722 patients; 295 ICU deaths) adjusted for age, SAPS II and SOFA, female sex was not associated with ICU mortality (odds ratio, 1.13; 95% CI, 0.81 to 1.57; p = 0.463) (Additional file 1: eTable 8).

For the primary mechanical-power analysis, the same Becher pressure-controlled-ventilation equation was applied uniformly. The PBW-normalised model included 410 patients contributing 44,725 observations. The female-sex coefficient was −0.01575 J·min⁻^1^·kg PBW⁻^1^ (95% CI, −0.05115 to 0.01964; p = 0.384), the height coefficient was −0.00389 J·min⁻^1^·kg PBW⁻^1^ per cm (95% CI, −0.00559 to −0.00220; p < 0.001), and the sex-by-height interaction was not supported (β = 0.0005; 95% CI, −0.0031 to 0.0042; p = 0.772). All variance inflation factors were ≤ 1.83.

Height-stratified female-sex coefficients for PBW-normalised mechanical power were −0.10415 J·min⁻^1^·kg PBW⁻^1^ below 165 cm (64 patients: 9 men and 55 women; 95% CI, −0.19483 to −0.01347; p = 0.028), 0.01114 at 165–175 cm (160 patients: 102 men and 58 women; 95% CI, −0.03250 to 0.05477; p = 0.618), and −0.02218 above 175 cm (186 patients: 178 men and 8 women; 95% CI, −0.10978 to 0.06542; p = 0.620). The extreme-stratum estimates should be interpreted cautiously because only nine men contributed below 165 cm and only eight women above 175 cm. In the absolute-MP sensitivity model, the female-sex coefficient was −2.64 J·min⁻^1^ (95% CI, −5.05 to −0.23; p = 0.032). In the restricted paired formula analysis (85 patients; 30,868 observations), the Gattinoni and Becher estimates were directionally concordant, but the female-sex coefficient in the model of the paired Gattinoni-minus-Becher difference was 0.0414 J·min⁻^1^·kg PBW⁻^1^ (95% CI, 0.0178 to 0.0650; p < 0.001), indicating formula-dependent magnitude (Additional file 1: eTables 6 and 7).

## Discussion

In this 11-year single-centre cohort of adults with ARDS, unadjusted analyses showed that women received higher PBW-normalised tidal volumes, had lower adherence to tidal-volume and driving-pressure targets, and had higher plateau and driving pressures than men. Women also had higher unadjusted PBW-normalised mechanical power, whereas absolute mechanical power was higher in men. In the simultaneous adjusted VT model, recorded height remained inversely associated with VT/kg PBW, but the female-sex coefficient was close to zero and imprecise; no sex-by-height interaction was detected. The corresponding primary mechanical-power model likewise did not support an independent association with recorded sex. These findings describe associations with recorded sex and recorded height and do not demonstrate mediation or identify inaccurate height acquisition as the underlying mechanism.

The lower tidal-volume adherence observed in women is consistent with the international LUNG SAFE cohort [8]. McNicholas and colleagues also found that women within the shorter subgroup (≤ 1.69 m) remained less likely than men to receive low tidal volumes after adjustment. In contrast, our height-stratified point estimates were closer to zero. The findings are not directly comparable: LUNG SAFE was a prospective multicentre study focused on the early ARDS period, whereas our analysis represents a single surgical ICU, used a longer observation window beginning at ICU admission, and applied different outcome definitions and height strata. Moreover, the wide confidence intervals in our extreme strata preclude concluding that a residual sex association is absent across the full height range.

Interpretation is complicated by PBW normalisation itself. PBW is calculated from both height and sex; therefore, the same absolute VT produces a higher VT/kg PBW in shorter patients and, at the same height, in women. The disparity may reflect incomplete scaling of clinician-set absolute VT to PBW, differences in respiratory physiology or case mix, inaccuracies in recorded height, or a combination of these mechanisms. In the absolute-VT sensitivity model, female sex was associated with a 30.1-mL lower adjusted VT and each additional centimetre of height with a 3.98-mL higher VT. Considered together with the PBW-normalised model, these results show that absolute delivered volume increased with recorded height but do not identify a causal mechanism or establish clinical benefit or harm.

Height documentation followed a stable operational process throughout the study period. At each new admission, the anaesthesiologist entered a new value as a mandatory field, most often during preoperative assessment, and previous values were not automatically carried forward. The acquisition field classified 48.4% of values as self-reported and 51.6% as determined by a physician; however, physician determination did not distinguish direct measurement from visual estimation, and proxy reports were not coded separately. The documented height-source category was not independently associated with VT/PBW and did not materially change the sex or height coefficients, but this analysis could not assess measurement accuracy because the physician-determined category combined direct measurement and visual estimation. Prospective studies have shown that visual estimation may be inaccurate, particularly in shorter patients [13, 14], making direct height measurement a plausible implementation target. Our data do not show that height inaccuracy caused the observed association or that direct measurement would eliminate it.

BMI and ventilator mechanics provide additional context. Women had a slightly higher median BMI (28.3 vs. 27.7 kg/m^2^; p = 0.053) and a higher prevalence of obesity (41.8% vs. 33.1%; p = 0.016). In the adjusted VT model, the BMI coefficient was 0.0145 mL/kg PBW per kg/m^2^ (95% CI, −0.0011 to 0.0302; p = 0.069), whereas BMI was positively associated with PBW-normalised mechanical power (β = 0.00365 J·min⁻^1^·kg PBW⁻^1^ per kg/m^2^; 95% CI, 0.00195 to 0.00535; p < 0.001). Obesity may alter chest-wall mechanics, airway pressures and ventilator settings, although PBW-based VT targets remain determined by height and sex rather than actual body weight. Women received a modestly lower PEEP. This cannot explain their higher VT/kg PBW; it may contribute arithmetically to higher driving pressure, whereas its direct contribution to calculated mechanical power would tend in the opposite direction. The uniform primary mechanical-power model did not support an independent sex association. In the restricted formula subset, both formula-specific estimates had the same direction, but their magnitude differed; this exploratory subset therefore does not change the primary inference and should not be interpreted as formula equivalence.

The present results do not establish which intervention should be preferred. Standardised direct height measurement, automatic PBW calculation and decision support are plausible candidates for prospective evaluation, particularly for shorter patients. The findings are hypothesis-generating and require validation in contemporary multicentre cohorts and prospective physiological or implementation studies that directly record the method and accuracy of height acquisition.

### Limitations

This study has several limitations. Its retrospective single-centre design in a surgical ICU limits generalisability and cannot separate local clinical culture from broader practice patterns. Height was not validated against a reference measurement; although documentation was mandatory and newly performed at each admission, the structured categories did not distinguish direct measurement from visual estimation or identify proxy reports separately. Ventilator data were analysed from ICU admission rather than a consistently reconstructed ARDS-onset time and may include pre-ARDS and recovery phases. Requiring at least 72 h of ventilation excluded patients who died or were liberated from ventilation earlier and may have introduced selection bias. Analyses were restricted to controlled ventilation, and differences in time spent in controlled modes may influence generalisability. Residual confounding remains possible despite expanded adjustment, particularly for changing physiology and clinical decisions over time. Missing covariate data reduced the adjusted cohort. Repeated high-frequency measurements may give greater weight to patients with longer ventilation; no patient-day aggregation sensitivity analysis was performed. The primary mechanical-power analysis used one uniform equation, whereas the comparison with the Gattinoni formulation was limited to a smaller, selected complete-case subset. Mortality analyses remain exploratory and should not be interpreted as establishing safety or causality.

## Conclusions

In this 11-year single-centre cohort, unadjusted analyses showed that women and shorter patients more frequently received tidal volumes above PBW-based lung-protective targets. In the simultaneous adjusted model, recorded height, but not recorded sex, was independently associated with VT/kg PBW; height-stratified estimates were imprecise where overlap between women and men was limited. Because PBW incorporates both sex and height and recorded height was not validated, the data cannot determine whether the unadjusted difference reflects clinician-set tidal volume, height documentation, respiratory physiology or other sex-associated factors. Standardised direct height measurement and PBW-based decision support are plausible targets for prospective evaluation.

## Supplementary Information


Additional file 1. Supplement — eFigures 1–5 and eTables 1–8, including triplet-combination adherence summaries, sensitivity analysis at the 4–6 mL/kg PBW threshold, ARDS-severity-stratified analyses, adjusted VT and mechanical-power models, the paired formula sensitivity analysis, and adjusted ICU mortality

## Data Availability

Deidentified individual patient-level data underlying the analyses, the data dictionary defining each field, and the statistical analysis code will be made available to investigators on reasonable request to the corresponding author, subject to approval by the Ethics Committee of the Medical Faculty of RWTH Aachen University and the institutional data-protection officer, and to execution of a data use agreement.

## Abbreviations

ARDS: Acute respiratory distress syndrome
BMI: Body mass index
CI: Confidence interval
DP: Driving pressure
ICD-10: International Statistical Classification of Diseases, Tenth Revision
ICU: Intensive care unit
IQR: Interquartile range
LPV: Lung-protective ventilation
MP: Mechanical power
PBW: Predicted body weight
PEEP: Positive end-expiratory pressure
Pplat: Plateau pressure
SAPS II: Simplified Acute Physiology Score II
SOFA: Sequential Organ Failure Assessment
STROBE: Strengthening the Reporting of Observational Studies in Epidemiology
VT: Tidal volume
