# Supplementary material for: Recorded body height and sex differences in lung-protective ventilation among adults with ARDS: an 11-year single-centre cohort study
Source: J Intensive Care. 2026 Aug 31;14:91. doi: 10.1186/s40560-026-00936-w (PMC13528038; doi:10.1186/s40560-026-00936-w)
Supplement: Supplementary file 1 [file 40560_2026_936_MOESM1_ESM.docx]

**Recorded body height and sex differences in lung-protective ventilation among adults with ARDS: an 11-year single-centre cohort study**

**Supplemental Digital Content**

Marius Huehn, MD¹; Lea Willemsen, MD¹; Matthias Manfred Deininger, MD¹; Judith Huth¹; Jana C. Mossanen, MD²; Jens Spiesshoefer, MD³; Gernot Marx, MD¹; Tim-Philipp Simon, MD¹; Thomas Breuer, MD¹

¹ Department of Intensive Care Medicine, Faculty of Medicine, RWTH Aachen University, Aachen, Germany
² Department of Anesthesiology, Faculty of Medicine, RWTH Aachen University, Aachen, Germany
³ Department of Pneumology and Intensive Care Medicine, Faculty of Medicine, RWTH Aachen University, Aachen, Germany

*This supplement provides additional figures and tables referenced in the main manuscript. All methodological detail and citations are contained in the main text; this document deliberately includes no separate text sections and no separate bibliography.*

**Contents**

eFigure 1 — Triplet combinations of LPV-adherent ventilator settings (first 30 days)

eFigure 2 — Triplet combinations of LPV-adherent ventilator settings (11-year period)

eFigure 3 — Sensitivity analysis at the 4–6 mL/kg PBW VT threshold

eFigure 4 — Ventilation parameters by sex and ARDS severity during the first 30 ICU days (4A–4C)

eFigure 5 — Ventilation parameters by sex and ARDS severity over the 11-year period (5A–5C)

eTable 1 — Triplet-evaluation of LPV adherence

eTable 2 — Linear mixed-effects models for tidal volume, including the height-source sensitivity analysis (2A–2D)

eTable 3 — LPV adherence stratified by ARDS severity (3A–3C)

eTable 4 — LPV adherence stratified by ARDS severity (4A–4C)

eTable 5 — Ventilation parameters over the 11-year period

eTable 6 — Linear mixed-effects models for mechanical power (6A–6C)

eTable 7 — Paired mechanical-power formula sensitivity analysis

eTable 8 — Adjusted ICU-mortality model

**eFigures**

eFigure 1. Triplet combinations of LPV-adherent ventilator settings during the first 30 days of ICU stay, by sex. Bars show the mean fraction (95 % CI, truncated at 0) of measurements in which 0, 1, 2 or 3 of the three primary LPV targets (VT, Pplat and DP) were met simultaneously. Women and men are colour- and icon-coded (♀/♂).

eFigure 2. Triplet combinations of LPV-adherent ventilator settings over the full 11-year period (2013–2023), by sex. Encoding is the same as in eFigure 1.

eFigure 3. Sensitivity analysis comparing the more protective 4–6 mL/kg PBW VT target with the ≤ 8 mL/kg PBW threshold during the first 30 ICU days. Sex-specific fractions of LPV-adherent measurements are shown; women and men are colour- and icon-coded (♀/♂).

eFigure 4A. Sex-specific course of DP (panel A), VT (panel B), Pplat (panel C) and PBW-normalised mechanical power (panel D) during the first 30 ICU days in mild ARDS (Berlin definition).

eFigure 4B. Sex-specific course of DP (panel A), VT (panel B), Pplat (panel C) and PBW-normalised mechanical power (panel D) during the first 30 ICU days in moderate ARDS (Berlin definition).

eFigure 4C. Sex-specific course of DP (panel A), VT (panel B), Pplat (panel C) and PBW-normalised mechanical power (panel D) during the first 30 ICU days in severe ARDS (Berlin definition).

eFigure 5A. Sex-specific course of DP (panel A), VT (panel B), Pplat (panel C) and PBW-normalised mechanical power (panel D) over the 11-year period in mild ARDS (Berlin definition).

eFigure 5B. Sex-specific course of DP (panel A), VT (panel B), Pplat (panel C) and PBW-normalised mechanical power (panel D) over the 11-year period in moderate ARDS (Berlin definition).

eFigure 5C. Sex-specific course of DP (panel A), VT (panel B), Pplat (panel C) and PBW-normalised mechanical power (panel D) over the 11-year period in severe ARDS (Berlin definition).

**eTables**

*eTable 1. Triplet evaluation of LPV adherence during the first 30 ICU days and the 11-year period. Women (♀) and men (♂). Values are mean (95 % CI). Combinations in which only DP, or only DP and VT, met the lung-protective threshold did not occur.*

| Triplet combination | Female (♀) mean (95 % CI) | Male (♂) mean (95 % CI) |
| --- | --- | --- |
| *First 30 days of ICU stay* |  |  |
| No LPV target met | 3.23 (1.96–4.5) % | 1.5 (0.88–2.12) % |
| Only VT | 5.33 (3.41–7.25) % | 6.14 (4.68–7.6) % |
| Only Pplat | 19.53 (16.38–22.68) % | 10.78 (8.92–12.64) % |
| Pplat and VT | 24.08 (20.53–27.63) % | 23.41 (20.68–26.14) % |
| Pplat and DP | 21.89 (18.37–25.41) % | 18.89 (16.67–21.11) % |
| All three LPV targets met | 25.93 (22.05–29.81) % | 39.29 (36.24–42.34) % |
| ***11-year period (2013–2023)*** |  |  |
| No LPV target met | 3.16 (1.93–4.39) % | 1.53 (0.92–2.14) % |
| Only VT | 5.69 (3.81–7.57) % | 6.48 (5.01–7.95) % |
| Only Pplat | 18.47 (15.55–21.39) % | 10.61 (8.83–12.39) % |
| Pplat and VT | 26.11 (22.46–29.76) % | 23.64 (20.98–26.3) % |
| Pplat and DP | 21.4 (18.01–24.79) % | 19.13 (16.95–21.31) % |
| All three LPV targets met | 25.17 (21.45–28.89) % | 38.61 (35.64–41.58) % |

*Included patients: ♀ 209, ♂ 413. CI, confidence interval; DP, driving pressure; LPV, lung-protective ventilation; Pplat, plateau pressure; VT, tidal volume.*

*eTable 2A. Primary adjusted linear mixed-effects model for PBW-normalised tidal volume (VT, mL/kg PBW). Fixed effects: recorded sex, recorded height centred at the mean of the VT complete-case analysis sample (174.1 cm), SAPS II, SOFA (maximum score within the first 24 hours after ICU admission), ARDS aetiology, age, BMI, linear relative ICU day and lowest same-day PaO₂/FiO₂ ratio; random intercept for patient; REML estimation. Complete-case sample: n = 527 patients; 58,802 observations.*

| **Variable** | **β** | **SE** | **95 % CI** | **t** | **p-value** |
| --- | --- | --- | --- | --- | --- |
| Intercept | 5.4995 | 0.3918 | 4.7316 – 6.2675 | 14.036 | < 0.001 |
| Female sex | 0.0514 | 0.1654 | −0.2728 – 0.3756 | 0.311 | 0.756 |
| Recorded height, per cm | −0.0342 | 0.0080 | −0.0498 – −0.0185 | −4.285 | < 0.001 |
| SAPS II | 0.0019 | 0.0061 | −0.0101 – 0.0139 | 0.311 | 0.756 |
| SOFA | −0.0175 | 0.0179 | −0.0525 – 0.0176 | −0.977 | 0.329 |
| ARDS aetiology: trauma | −0.8180 | 0.1303 | −1.0735 – −0.5626 | −6.276 | < 0.001 |
| Age, per year | 0.0098 | 0.0047 | 0.0005 – 0.0191 | 2.072 | 0.039 |
| BMI, per kg/m² | 0.0145 | 0.0080 | −0.0011 – 0.0302 | 1.822 | 0.069 |
| Relative ICU day, linear | 0.0319 | 0.0010 | 0.0300 – 0.0339 | 31.521 | < 0.001 |
| PaO₂/FiO₂, per mmHg | 0.0021 | 0.0001 | 0.0019 – 0.0023 | 20.307 | < 0.001 |

β values are adjusted mean differences in mL/kg PBW. Reference groups were male sex and non-traumatic ARDS aetiology. CI, confidence interval; PBW, predicted body weight; REML, restricted maximum likelihood; SE, standard error; VT, tidal volume. All variance inflation factors were ≤ 1.75.

*eTable 2B. Female-sex coefficient across sequential PBW-normalised VT models and in the absolute-VT sensitivity model.*

| **Model / outcome** | **Female-sex β** | **95 % CI** | **p-value** |
| --- | --- | --- | --- |
| M0: SAPS II, SOFA, ARDS aetiology | 0.5373 mL/kg PBW | 0.2846 – 0.7899 | < 0.001 |
| M1: M0 + age, BMI, ICU day, PaO₂/FiO₂ | 0.5022 mL/kg PBW | 0.2482 – 0.7563 | < 0.001 |
| M2: M1 + recorded height | 0.0514 mL/kg PBW | −0.2728 – 0.3756 | 0.756 |
| M3: M2 + sex × height | 0.0549 mL/kg PBW | −0.3227 – 0.4325 | 0.776 |
| Absolute VT sensitivity, mL | −30.1193 mL | −51.8781 – −8.3605 | 0.007 |

*eTable 2C. Adjusted height-stratified linear mixed-effects analyses of PBW-normalised VT. Each model used the M1 covariates and a patient-level random intercept. β represents the adjusted female-versus-male mean difference within each stratum.*

| **Height stratum** | **Patients, n (men/women)** | **β, mL/kg PBW** | **SE** | **95 % CI** | **p-value** |
| --- | --- | --- | --- | --- | --- |
| < 165 cm | 87 (14/73) | −0.055 | 0.445 | −0.927 – 0.817 | 0.902 |
| 165–175 cm | 213 (135/78) | 0.067 | 0.198 | −0.322 – 0.455 | 0.737 |
| > 175 cm | 227 (215/12) | −0.109 | 0.387 | −0.868 – 0.650 | 0.779 |

*eTable 2D. Adjusted linear mixed-effects model for PBW-normalised tidal volume additionally including the documented height-source category. Fixed effects: recorded sex, recorded height centred at the mean of the VT complete-case analysis sample (174.1 cm), SAPS II, SOFA (maximum score within the first 24 hours after ICU admission), ARDS aetiology, age, BMI, linear relative ICU day, lowest same-day PaO₂/FiO₂ ratio and documented height-source category; random intercept for patient; REML estimation. Complete-case sample: n = 527 patients; 58,802 observations.*

| **Variable** | **β** | **SE** | **95 % CI** | **t** | **p-value** |
| --- | --- | --- | --- | --- | --- |
| Intercept | 5.5504 | 0.3982 | 4.7700 – 6.3308 | 13.940 | < 0.001 |
| Female sex | 0.0513 | 0.1655 | −0.2732 – 0.3757 | 0.310 | 0.757 |
| Recorded height, per cm | −0.0338 | 0.0080 | −0.0495 – −0.0182 | −4.232 | < 0.001 |
| SAPS II | 0.0016 | 0.0061 | −0.0104 – 0.0137 | 0.268 | 0.789 |
| SOFA | −0.0165 | 0.0179 | −0.0517 – 0.0186 | −0.922 | 0.357 |
| ARDS aetiology: trauma | −0.8106 | 0.1308 | −1.0670 – −0.5542 | −6.196 | < 0.001 |
| Age, per year | 0.0096 | 0.0048 | 0.0003 – 0.0189 | 2.013 | 0.045 |
| BMI, per kg/m² | 0.0149 | 0.0080 | −0.0008 – 0.0306 | 1.862 | 0.063 |
| Relative ICU day, linear | 0.0319 | 0.0010 | 0.0299 – 0.0339 | 31.518 | < 0.001 |
| PaO₂/FiO₂, per mmHg | 0.0021 | 0.0001 | 0.0019 – 0.0023 | 20.306 | < 0.001 |
| Documented height source: determined by physician | −0.0874 | 0.1196 | −0.3217 – 0.1470 | −0.731 | 0.465 |

β values are adjusted mean differences in mL/kg PBW. Reference groups were male sex, non-traumatic ARDS aetiology and self-reported height source. The height-source coefficient compares physician-determined with self-reported height. CI, confidence interval; PBW, predicted body weight; REML, restricted maximum likelihood; SE, standard error; VT, tidal volume.

*eTable 3A. LPV adherence — mild ARDS subgroup, first 30 ICU days.*

| Value | Patients (n) | Median [IQR] | Mean ± SD | LPV fraction, mean ± SD, % | p-value |
| --- | --- | --- | --- | --- | --- |
| VT, ♀ (mL/kg PBW) | 179 | 7.7 [6.7–8.4] | 7.6 ± 1.4 | 55.2 ± 32.1 | p = 0.001 |
| VT, ♂ (mL/kg PBW) | 371 | 7.2 [6.4–7.9] | 7.2 ± 1.4 | 64.9 ± 29.9 | p = 0.001 |
| Pplat, ♀ (cm H₂O) | 155 | 22.4 [19.3–25.1] | 22.1 ± 4.0 | 97.5 ± 10.6 | p = 0.992 |
| Pplat, ♂ (cm H₂O) | 291 | 20.9 [18.1–23.8] | 21.0 ± 4.0 | 96.7 ± 13.0 | p = 0.992 |
| DP, ♀ (cm H₂O) | 127 | 13.6 [11.7–15.7] | 13.6 ± 2.8 | 52.8 ± 41.3 | p < 0.001 |
| DP, ♂ (cm H₂O) | 264 | 12.2 [10.2–14.5] | 12.5 ± 3.5 | 67.5 ± 38.4 | p < 0.001 |

*eTable 3B. LPV adherence — moderate ARDS subgroup, first 30 ICU days.*

| Value | Patients (n) | Median [IQR] | Mean ± SD | LPV fraction, mean ± SD, % | p-value |
| --- | --- | --- | --- | --- | --- |
| VT, ♀ (mL/kg PBW) | 218 | 7.6 [6.6–8.3] | 7.4 ± 1.7 | 56.3 ± 31.9 | p < 0.001 |
| VT, ♂ (mL/kg PBW) | 447 | 6.8 [6.1–7.6] | 6.8 ± 1.4 | 70.2 ± 27.7 | p < 0.001 |
| Pplat, ♀ (cm H₂O) | 197 | 24.0 [21.4–26.8] | 24.0 ± 4.1 | 90.5 ± 21.8 | p = 0.056 |
| Pplat, ♂ (cm H₂O) | 376 | 23.5 [20.4–26.0] | 23.3 ± 4.1 | 92.9 ± 19.1 | p = 0.056 |
| DP, ♀ (cm H₂O) | 174 | 14.3 [12.5–16.3] | 14.3 ± 3.2 | 44.7 ± 40.6 | p = 0.023 |
| DP, ♂ (cm H₂O) | 329 | 13.4 [11.4–15.3] | 13.5 ± 3.2 | 54.0 ± 39.8 | p = 0.023 |

*eTable 3C. LPV adherence — severe ARDS subgroup, first 30 ICU days.*

| Value | Patients (n) | Median [IQR] | Mean ± SD | LPV fraction, mean ± SD, % | p-value |
| --- | --- | --- | --- | --- | --- |
| VT, ♀ (mL/kg PBW) | 124 | 7.2 [6.3–8.0] | 7.0 ± 1.9 | 57.5 ± 33.7 | p = 0.024 |
| VT, ♂ (mL/kg PBW) | 265 | 6.7 [5.8–7.4] | 6.6 ± 1.9 | 65.5 ± 32.9 | p = 0.024 |
| Pplat, ♀ (cm H₂O) | 106 | 25.9 [22.1–28.6] | 25.4 ± 4.7 | 81.1 ± 34.1 | p = 0.572 |
| Pplat, ♂ (cm H₂O) | 203 | 24.1 [20.9–28.0] | 24.4 ± 4.6 | 84.9 ± 28.7 | p = 0.572 |
| DP, ♀ (cm H₂O) | 95 | 14.3 [12.2–16.7] | 14.6 ± 3.7 | 46.8 ± 43.1 | p = 0.452 |
| DP, ♂ (cm H₂O) | 181 | 13.7 [11.5–15.7] | 14.0 ± 3.8 | 50.4 ± 41.8 | p = 0.452 |

*Data are presented as median [IQR] or mean ± SD. CI, confidence interval; DP, driving pressure; IQR, interquartile range; LPV, lung-protective ventilation; PBW, predicted body weight; Pplat, plateau pressure; SD, standard deviation; VT, tidal volume. ARDS severity follows the Berlin definition (mild: PaO₂/FiO₂ 201 – 300 mmHg; moderate: 101 – 200 mmHg; severe: ≤ 100 mmHg).*

*eTable 4A. LPV adherence — mild ARDS subgroup, 11-year period.*

| Value | Patients (n) | Median [IQR] | Mean ± SD | LPV fraction, mean ± SD, % | p-value |
| --- | --- | --- | --- | --- | --- |
| VT, ♀ (mL/kg PBW) | 188 | 7.5 [6.7–8.4] | 7.5 ± 1.4 | 56.4 ± 31.5 | p = 0.004 |
| VT, ♂ (mL/kg PBW) | 386 | 7.1 [6.4–7.9] | 7.2 ± 1.4 | 64.7 ± 29.5 | p = 0.004 |
| Pplat, ♀ (cm H₂O) | 167 | 22.3 [19.4–25.2] | 22.2 ± 4.0 | 97.1 ± 10.6 | p = 0.265 |
| Pplat, ♂ (cm H₂O) | 308 | 20.9 [18.0–23.5] | 20.9 ± 4.0 | 96.6 ± 12.9 | p = 0.265 |
| DP, ♀ (cm H₂O) | 139 | 13.8 [11.8–15.9] | 13.8 ± 2.9 | 51.5 ± 41.0 | p < 0.001 |
| DP, ♂ (cm H₂O) | 278 | 12.2 [10.2–14.4] | 12.5 ± 3.5 | 68.3 ± 37.8 | p < 0.001 |

*eTable 4B. LPV adherence — moderate ARDS subgroup, 11-year period.*

| Value | Patients (n) | Median [IQR] | Mean ± SD | LPV fraction, mean ± SD, % | p-value |
| --- | --- | --- | --- | --- | --- |
| VT, ♀ (mL/kg PBW) | 225 | 7.5 [6.6–8.4] | 7.3 ± 1.7 | 57.1 ± 31.2 | p < 0.001 |
| VT, ♂ (mL/kg PBW) | 462 | 6.8 [6.0–7.5] | 6.7 ± 1.4 | 70.1 ± 27.5 | p < 0.001 |
| Pplat, ♀ (cm H₂O) | 203 | 24.3 [21.6–26.9] | 24.1 ± 4.0 | 90.2 ± 20.8 | p = 0.021 |
| Pplat, ♂ (cm H₂O) | 393 | 23.5 [20.4–26.0] | 23.3 ± 4.1 | 92.5 ± 19.4 | p = 0.021 |
| DP, ♀ (cm H₂O) | 180 | 14.3 [12.7–16.3] | 14.5 ± 3.1 | 43.4 ± 39.4 | p = 0.013 |
| DP, ♂ (cm H₂O) | 342 | 13.5 [11.5–15.3] | 13.6 ± 3.3 | 53.1 ± 39.6 | p = 0.013 |

*eTable 4C. LPV adherence — severe ARDS subgroup, 11-year period.*

| Value | Patients (n) | Median [IQR] | Mean ± SD | LPV fraction, mean ± SD, % | p-value |
| --- | --- | --- | --- | --- | --- |
| VT, ♀ (mL/kg PBW) | 131 | 7.2 [6.2–8.1] | 7.1 ± 1.8 | 57.5 ± 33.1 | p = 0.013 |
| VT, ♂ (mL/kg PBW) | 287 | 6.6 [5.77–7.4] | 6.5 ± 1.8 | 66.1 ± 31.6 | p = 0.013 |
| Pplat, ♀ (cm H₂O) | 113 | 25.6 [22.2–29.1] | 25.5 ± 4.6 | 80.7 ± 34.0 | p = 0.396 |
| Pplat, ♂ (cm H₂O) | 226 | 24.3 [20.9–28.0] | 24.4 ± 4.8 | 83.9 ± 29.7 | p = 0.396 |
| DP, ♀ (cm H₂O) | 101 | 14.9 [12.2–16.9] | 14.9 ± 3.7 | 42.9 ± 41.8 | p = 0.240 |
| DP, ♂ (cm H₂O) | 201 | 13.8 [11.6–16.3] | 14.1 ± 3.9 | 48.7 ± 41.8 | p = 0.240 |

*Data are presented as median [IQR] or mean ± SD. CI, confidence interval; DP, driving pressure; IQR, interquartile range; LPV, lung-protective ventilation; PBW, predicted body weight; Pplat, plateau pressure; SD, standard deviation; VT, tidal volume. ARDS severity follows the Berlin definition (mild: PaO₂/FiO₂ 201 – 300 mmHg; moderate: 101 – 200 mmHg; severe: ≤ 100 mmHg).*

*eTable 5. Ventilation parameters over the 11-year period, by sex.*

| Value (units) | Female n | Female median [IQR] | Female mean ± SD | Male n | Male median [IQR] | Male mean ± SD | p-value |
| --- | --- | --- | --- | --- | --- | --- | --- |
| VT, mL/kg PBW | 260 | 7.5 [6.7–8.3] | 7.4 ± 1.5 | 525 | 6.8 [6.2–7.5] | 6.7 ± 1.3 | < 0.001 |
| VT adherence fraction (≤ 8 mL/kg PBW), % | 260 | 56.5 [37–77.8] | 56.1 ± 26.5 | 525 | 76 [59–88.3] | 70.6 ± 22.5 | < 0.001 |
| Pplat, cm H₂O | 251 | 23.9 [21.5–26.7] | 23.9 ± 3.7 | 494 | 22.5 [20.2–25.5] | 22.8 ± 3.9 | < 0.001 |
| Pplat adherence fraction (≤ 30 cm H₂O), % | 251 | 100 [89.3–100] | 90.3 ± 17.9 | 494 | 100 [94.4–100] | 92.3 ± 17.4 | 0.013 |
| DP, cm H₂O | 222 | 14.0 [12.3–16.1] | 14.3 ± 2.8 | 448 | 13.0 [11.2–15.0] | 13.2 ± 3.1 | < 0.001 |
| DP adherence fraction (< 15 cm H₂O), % | 222 | 41.5 [9.4–81.6] | 46.2 ± 36.4 | 448 | 64.2 [20.6–95.5] | 57.4 ± 36.6 | < 0.001 |
| Absolute MP, J·min⁻¹ | 203 | 22.8 [18.7–27.8] | 23.1 ± 7.7 | 437 | 27.0 [21.9–32.2] | 27.5 ± 9.0 | < 0.001 |
| PBW-normalised MP, J·min⁻¹·kg PBW⁻¹ | 203 | 0.40 [0.34–0.50] | 0.41 ± 0.14 | 437 | 0.37 [0.30–0.45] | 0.38 ± 0.13 | < 0.001 |

*Data are presented as median [IQR] or mean ± SD. CI, confidence interval; DP, driving pressure; IQR, interquartile range; LPV, lung-protective ventilation; MP, mechanical power; PBW, predicted body weight; Pplat, plateau pressure; SD, standard deviation; VT, tidal volume.*

*eTable 6A. Primary adjusted linear mixed-effects model for PBW-normalised mechanical power.*

Fixed effects: recorded sex, recorded height centred at the mean of the mechanical-power complete-case analysis sample (174.46 cm), SAPS II, SOFA (maximum score within the first 24 hours after ICU admission), ARDS aetiology, age, BMI, linear relative ICU day and lowest same-day PaO₂/FiO₂ ratio; random intercept for patient. The same Becher pressure-controlled-ventilation equation was applied to every patient. Complete-case sample: n = 410 patients; 44,725 observations.

| **Variable** | **β** | **SE** | **95 % CI** | **p-value** |
| --- | --- | --- | --- | --- |
| Intercept | 0.30206 | 0.04138 | 0.22095 – 0.38317 | < 0.001 |
| Female sex | −0.01575 | 0.01806 | −0.05115 – 0.01964 | 0.384 |
| Recorded height, per cm | −0.00389 | 0.00087 | −0.00559 to −0.00220 | < 0.001 |
| SAPS II | 0.00021 | 0.00066 | −0.00107 – 0.00150 | 0.745 |
| SOFA | −0.00047 | 0.00181 | −0.00402 – 0.00308 | 0.796 |
| ARDS aetiology: trauma | −0.00780 | 0.01370 | −0.03466 – 0.01905 | 0.569 |
| Age, per year | 0.00002 | 0.00049 | −0.00093 – 0.00098 | 0.960 |
| BMI, per kg/m² | 0.00365 | 0.00087 | 0.00195 – 0.00535 | < 0.001 |
| Relative ICU day, linear | 0.00236 | 0.00010 | 0.00217 – 0.00256 | < 0.001 |
| PaO₂/FiO₂, per mmHg | −0.00029 | 0.00001 | −0.00031 – −0.00027 | < 0.001 |

β values are adjusted mean differences in J·min⁻¹·kg PBW⁻¹. Reference groups were male sex and non-traumatic ARDS aetiology. All variance inflation factors were ≤ 1.83.

*eTable 6B. Height-stratified adjusted models for PBW-normalised mechanical power.*

| **Height stratum** | **Patients, n (men/women)** | **Female-sex β** | **95 % CI** | **p-value** |
| --- | --- | --- | --- | --- |
| < 165 cm | 64 (9/55) | −0.10415 | −0.19483 – −0.01347 | 0.028 |
| 165–175 cm | 160 (102/58) | 0.01114 | −0.03250 – 0.05477 | 0.618 |
| > 175 cm | 186 (178/8) | −0.02218 | −0.10978 – 0.06542 | 0.620 |

β values are adjusted female-versus-male mean differences in J·min⁻¹·kg PBW⁻¹. Models used the eTable 6A covariates except height. The shortest and tallest strata have limited sex overlap.

*eTable 6C. Adjusted absolute-mechanical-power sensitivity model.*

| **Variable** | **β, J·min⁻¹** | **SE** | **95 % CI** | **p-value** |
| --- | --- | --- | --- | --- |
| Intercept | 21.30852 | 2.81862 | 15.78413 to 26.83290 | < 0.001 |
| Female sex | −2.64374 | 1.22994 | −5.05438 to −0.2330 | 0.032 |
| Recorded height, per cm | 0.10998 | 0.05893 | −0.00552 – 0.22549 | 0.063 |
| SAPS II | 0.01997 | 0.04463 | −0.06750 – 0.10744 | 0.655 |
| SOFA | −0.03534 | 0.12341 | −0.27722 – 0.20654 | 0.775 |
| ARDS aetiology: trauma | −0.52733 | 0.93297 | −2.356 – 1.301 | 0.572 |
| Age, per year | 0.00369 | 0.03321 | −0.061 – 0.069 | 0.912 |
| BMI, per kg/m² | 0.21940 | 0.05909 | 0.104 – 0.335 | < 0.001 |
| Relative ICU day, linear | 0.15975 | 0.00686 | 0.146 – 0.173 | < 0.001 |
| PaO₂/FiO₂, per mmHg | −0.01849 | 0.00072 | −0.020 – −0.017 | < 0.001 |

*eTable 7. Exploratory paired mechanical-power formula sensitivity analysis.*

Of 166 patients with the variables required for the Gattinoni formulation, 146 also had temporally overlapping estimates from the Becher pressure-controlled-ventilation equation. Complete covariate data at identical valid time points were available for 85 patients (60 men and 25 women; 30,868 observations). This restricted subset differs from the primary mechanical-power cohort; the analysis is exploratory and is not used for the main inference.

| **Outcome** | **Patients, n (men/women)** | **Female-sex β** | **95 % CI** | **p-value** |
| --- | --- | --- | --- | --- |
| Gattinoni, PBW-normalised, J·min⁻¹·kg PBW⁻¹ | 85 (60/25) | −0.08829 | −0.17 – −0.01 | 0.040 |
| Becher PCV, PBW-normalised, J·min⁻¹·kg PBW⁻¹ | 85 (60/25) | −0.13397 | −0.23 – −0.04 | 0.009 |
| Paired Gattinoni − Becher PCV, PBW-normalised | 85 (60/25) | 0.04144 | 0.02 – 0.07 | < 0.001 |
| Gattinoni, absolute, J·min⁻¹ | 85 (60/25) | −6.78973 | −12.59 – −0.99 | 0.024 |
| Becher PCV, absolute, J·min⁻¹ | 85 (60/25) | −9.92574 | −16.76 – −3.09 | 0.006 |
| Paired Gattinoni − Becher PCV, absolute | 85 (60/25) | 2.85165 | 1.18 – 4.52 | 0.001 |

Models included recorded sex, centred recorded height, SAPS II, SOFA (maximum score within the first 24 hours after ICU admission), ARDS aetiology, age, BMI, linear relative ICU day and the lowest same-day PaO₂/FiO₂ ratio, with a patient-level random intercept. For the paired-difference outcomes, the dependent variable was the within-time-point Gattinoni estimate minus the Becher estimate; a positive female-sex coefficient therefore indicates a larger formula difference in women. The significant paired-difference coefficients show that formula choice affected estimated magnitude. Sex distribution by height stratum was < 165 cm, 1 man and 11 women; 165–175 cm, 20 men and 13 women; and > 175 cm, 39 men and 1 woman. Height-stratified models were therefore unstable in the extreme strata.

eTable 8. Exploratory adjusted logistic-regression model for ICU mortality.

| **Variable** | **Odds ratio** | **95 % CI** | **p-value** |
| --- | --- | --- | --- |
| Female sex | 1.13 | 0.81 – 1.57 | 0.463 |
| Age, per year | 1.03 | 1.01 – 1.04 | < 0.001 |
| SAPS II | 1.02 | 1.00 to 1.04 | 0.014 |
| SOFA | 1.03 | 0.98 – 1.08 | 0.244 |

Complete-case sample: n = 722 patients; 295 ICU deaths (40.9 %). Reference group for sex: male. SOFA was the maximum score within the first 24 hours after ICU admission. Variance inflation factors ranged from 1.004 to 1.394. CI, confidence interval; SAPS II, Simplified Acute Physiology Score II; SOFA, Sequential Organ Failure Assessment.
